# A candidate probiotic strain of *Enterococcus faecium* from the intestine of the crucian carp *Carassius auratus*

**DOI:** 10.1186/s13568-020-00973-0

**Published:** 2020-02-27

**Authors:** Qing Mao, Xueliang Sun, Jingfeng Sun, Feng Zhang, Aijun Lv, Xiucai Hu, Yongjun Guo

**Affiliations:** Tianjin Key Lab of Aqua-ecology and Aquaculture, Fisheries College, Tianjin Agricultural University, Tianjin, 300384 China

**Keywords:** *Carassius auratus*, *Enterococcus faecium*, 16S rDNA, Bacteriostatic experiment, Growth characteristics

## Abstract

In the present study, a Gram-positive bacterium was isolated from the intestine of healthy crucian carp *Carassius auratus* and named strain R8. It was initially identified as *Enterococcus faecium* according to its morphological, physiological and biochemical characteristics. Further identification by using 16S rRNA gene sequence analysis confirmed the R8 strain (Genbank accession no. MF928076) as *E. faecium.* Challenge and hemolysis experiments showed that the *E. faecium* R8 strain had no toxicity to the crucian carp. Bacteriostatic experiment showed that this isolate obviously inhibited the growth of *Aeromonas veronii* and *Staphylococcus haemolyticus.* The proliferation of *E. faecium* R8 strain occurred after exposure to various growth conditions such as at pH values from 2.0 to 4.0 for 8 h, bile concentrations from 0.2 to 1.2% and high temperature of 80 °C. This bacterial strain grew best under the condition of 37 °C, pH 7.0 and salinity 30 ppt, and its growth curve exhibited four distinct phases. These results showed that the *E. faecium* R8 strain had potential probiotic characteristics and could be used as a candidate strain for aquatic probiotics.

## Introduction

The crucian carp *Carassius auratus,* one of the important freshwater economic fish, occupies an important position in China’s aquaculture industry (Wu et al. 2015). With the rapid development of intensive culture with high density, *C. auratus* has been subject to various bacterial diseases, which caused great economic losses (Ostland et al. 1989; Li et al. 2017). Although antibiotics play an important role in the treatment of fish bacterial disease, their application are limited because of the emergence of drug resistance of certain pathogens, and the potential damage to their hosts and human beings (Laxminarayan et al. 2013).

In recent years, it has aroused considerable interests to use some probiotic microorganisms in feeds as an alternative to antibiotics to prevent various diseases (Guerra et al. 2007). Probiotics are live microbial feed supplements that inhibit the growth of pathogenic bacteria by adhering to and colonizing animal intestines, competing for nutrients and space and producing antimicrobial substances (Watson et al. 2008). Moreover, they have beneficial effects on the host by improving its intestinal microbial balance (Suzer et al. 2008). Therefore, probiotics have received extensive attention in the aquaculture industry and become a hot topic in the research area of aquatic disease prevention or control.

*Enterococcus* is the core intestinal flora of humans and widespread in the intestines of most animals (Lebreton et al. 2017; Schloissnig et al. 2013; Van Tyne and Gilmore 2014). Some species of *Enterococcus* were used as probiotics in many countries because of the high ability to produce bacteriocins (Franz et al. 2003). *Enterococcus faecium*, an important species of *Enterococcus*, has ever been isolated from numerous sources, such as dairy products, shrimp and mammalian gastrointestinal tracts (Franz et al. 1996; Swain et al. 2009; Liu et al. 2017). *E. faecium* has many biological characteristics, such as surviving in the environment of strong acid and high concentration of bile salt and inhibiting the growth of pathogenic bacteria (Saelim et al. 2012; Lin et al. 2007). Subsequently, it has been widely used in the feed additive industry as a probiotic (Foulquié Moreno et al. 2006). Currently, *E. faecium* used in aquaculture is mostly derived from terrestrial animal or commercial preparation of unknown origin (Liu et al. 2011). There are many reports on the positive effects of these preparations for aquatic animal, such as increasing the final body weight (FBW) and daily weight (DWG), enhancing the serum complement activity, and improving the phagocytosis function of macrophages (Wang et al. 2008; Panigrahi et al. 2007; Ju et al. 2018). However, previous studies have showed that if a probiotic strain was not derived from the host itself, it might not be able to colonize effectively in its body and function as expected, subsequently acting as only a placebo (Lazado et al. 2010). Therefore, it is important to seek for suitable native bacteria from aquatic animals to be used for aquatic probiotics.

There are few reports about the utilization of *E. faecium* strains isolated from aquatic animals as probiotics (Gopalakannan and Arul 2011; Zeng et al. 2009). In the present study, a Gram-positive bacterium was isolated from the intestinal tract of healthy *C. auratus* and identified as *E. faecium* according to its physiological and biochemical characteristics and genotype. Potential probiotic properties of *E. faecium*, such as antagonistic activity, tolerant ability against various physical and chemical factors and safety characteristics were studied.

## Materials and methods

### Isolation of bacteria

The bacterial strains were isolated from the intestinal tract of healthy *C. auratus* (n = 10, 200–220 g) and cultured on de Man-Rogosa-Sharpe (MRS) agar plates (Beijing Solarbio Science and Technology Co., Ltd., Beijing, China) at 37 °C for 24 h. Colonies differing in morphological characteristics were selected and subcultured in MRS broth. A total of eight bacterial strains from different colonies were isolated, purified and stored in sterile glycerol (15% v/v) at − 80 °C.

### Antagonistic activity

Four indicator pathogens were used in this study, including *Aeromonas veronii*, S*taphylococcus haemolyticus*, *Vibrio parahaemolyticus* and *Vibrio vulnificus*, stored in the Key Lab of Aqua-ecology and Aquaculture, Tianjin Agricultural University. The concentrations of the eight isolated strains and the indicator pathogens were adjusted to 1 × 10^6^ CFU/mL and 1 × 10^7^ CFU/mL, respectively. Antagonistic activities of the eight isolated bacterial strains against these indicator pathogens were determined according to the Oxford cup method (Vincent et al. 1944). In brief, 100 μL of bacterial suspension of each indicator pathogen was spread evenly on a Luria–Bertani (LB) agar plates (tryptone 10 g/L, yeast extract powder 5 g/L, agar 15 g/L and Nacl 10 g/L; Beijing Aoboxing Bio-Technology Co., Ltd., Beijing, China) and allowed to absorb, then equal volume of bacterial suspension of the isolate to be tested was placed in the Oxford cups. The LB agar plate was incubated at 37 °C for 24 h, then the antagonistic activity was examined according to the diameters of inhibition zones appearing around the cups. A strain named R8 with stronger inhibitory activity against the pathogenic bacteria was selected for the subsequent experiments.

### Biochemical characteristics tests

The Gram-staining method was used for the morphological investigation. The commercial microtest systems (Hangzhou Tianhe Microorganism Reagent Co., Ltd., Hangzhou, China) were used to perform the biochemical tests, including oxidative/fermentative, methyl red test, urea, Voges Proskauer test, gluconate, catalase, oxidase, arginine, NO_3_^−^ reductase, amylum, sorbitol, mannitol, saligenin, sucrose, raffinose, glucose, xylose, lactose, bile esculin and arabinose. And it was also studied for the growth condition at 0–10% of NaCl (w/v) and temperature of 4–42 °C. The incubation was performed at 37 °C for 48 h and the results were observed with reference to the manual of systematic and determinative bacteriology (Dong and Cai 2001).

### Genotypic identification

The boiling method was used to extract total genomic DNA of the isolate (Chen et al. 2015). The 16S rRNA gene was amplified with a pair of universal primers, 27F: 5′-AGAGTTTGATCATGGCTCAG-3′ and 1492R: 5′-GGTTACCTTGTTACGACTT-3′ (Cao et al. 2007). The PCR reaction of the samples underwent an initial denaturation of 4 min at 95 °C, and then 30 cycles of 45 s at 94 °C, 45 s at 55 °C and 1 min at 72 °C, followed by 10 min at 72 °C. Reaction products were purified and cloned according to the report of Han et al. (2017). The nucleotide sequences were compared with known sequences in the NCBI database by using the Blast tool (NCBI, http://www.ncbi.nih.gov/BLAST/). The neighbor-joining algorithm of MEGA 5.22 software was used to construct the phylogenetic trees, with 1000 bootstrap replicates.

### Haemolysis experiment

According to the method of Yang et al. (2013), the haemolytic analysis of the bacterial isolate R8 was performed on a blood agar plate.

### Pathogenicity test

Sixty healthy *C. auratus* with an average weight of 74 ± 10 g and length of 14 ± 0.5 cm were purchased from a large aquatic wholesale market in Tianjin, China. Fish were transferred back to the Tianjin Agricultural University and acclimatized for 2 weeks, with water temperature adjusted to 28 °C and pH 7.5. Aeration was provided to maintain optimal DO and fish were fed with commercial feed pellets twice daily. All the fish were randomly divided into five groups with twelve fish in each group. Four groups were injected intraperitoneally with 0.2 mL of the suspension of R8 strain at a concentration of 1 × 10^5^ CFU/mL, 1 × 10^6^ CFU/mL, 1 × 10^7^ CFU/mL and 1 × 10^8^ CFU/mL, respectively. The last group used as control was injected with the same dose of 0.85% physiological saline. The health condition and mortality of *C. auratus* were observed within 14 days after injection. The protocol was approved by the Animal Experimentation Ethical Committee of the Tianjin Agricultural University.

### Tolerant ability against acid, bile and temperature

The tolerant abilities against various pH value, bile conditions and temperature were determined. The bacterial isolate R8 stored in glycerol-cryopreservative medium was resuscitated in MRS broth at 37 °C until arriving at stationary phase. Then the bacterial suspension was adjusted to a concentration of 1 × 10^8^ CFU/mL. To determine the acid tolerance of this bacterial isolate, 0.5 mL of the bacterial suspension was inoculated into 10 mL of LB broth with pH values of 2, 3 or 4, and cultured for 2 h, 4 h and 8 h, respectively at 37 °C, and the value of optical density (OD) was read at 600 nm with a ultraviolet and visible spectrophotometer (Beijing Purkinje General Instrument Co., Ltd., Beijing, China). The pH value of LB broth was adjusted with the sterile solution of 1 mol/L NaOH or 1 mol/L HCl.

For determination of the tolerance against bile, 0.5 mL of the R8 bacterial suspension (1 × 10^8^ CFU/mL) was inoculated into 10 mL of LB broth supplemented with 0.2%, 0.4%, 0.6%, 0.8%, 1.0% and 1.2% bile salts or without (control), and incubated for 24 h at 37 °C. Then, the value of OD of the bacterial suspension at 600 nm was recorded.

To test the tolerance against various temperatures, 0.5 mL of the R8 bacterial suspension was inoculated into 10 mL of LB broth and the temperature was controlled at 50 °C, 60 °C, 70 °C, 80 °C and 90 °C. Growth was checked at 2 min, 5 min, 10 min, 20 min and 30 min, respectively and the value of OD of the bacterial suspension at 600 nm was recorded.

### Growth characteristics

Growth characteristics were tested according to the method of Han et al. (2017). The temperature, pH value and NaCl concentration were adjusted to various conditions based on LB broth (Beijing Aoboxing Bio-Technology Co., Ltd., Beijing, China). The impact of pH value of 4, 5, 6, 7, 8 and 9 on the growth of the bacterial isolate R8 was studied in LB broth with 30 ppt at 37 °C; the impact of salinity of 20 ppt, 30 ppt and 40 ppt was studied in LB broth with pH value of 7.0 at 37 °C; the impact of temperature at 27 °C, 32 °C, 37 °C and 42 °C was studied in LB broth with the pH value of 7.0 at 30 ppt. The LB broth for bacterial cultivation was inoculated with 200 μL bacterial suspension with a concentration of 1 × 10^8^ CFU/mL, and incubated at 180 rpm for 28 h. The OD values at 600 nm of the bacterial suspensions were measured every 2 h.

## Results

### Isolation and biochemical characteristics

A bacterial strain with bacteriostatic ability was isolated and screened from the intestinal tract of healthy *C. auratus* and named R8. It was Gram-positive and its colonies on MRS plate were circular, smooth and ivory in colour after incubation for 24 h at 37 °C. It could grow at 42 °C and within a concentration range of 0–10% NaCl (w/v). The biochemical results were showed in Table 1. The isolate was oxidized, able to hydrolyse urea, bile esculin and amylum, reduce nitrate to nitrite, but unable to utilize gluconate. Furthermore, it was positive for methyl red test, production of arginine hydrolase and oxidase, acid production from arabinose, mannitol, sucrose, saligenin, glucose, lactose, but negative for catalase, Voges Proskauer test, and acid production from sorbitol, gluconate, raffinose and xylose.

**Table 1:** Biochemical characteristics of the R8 strain

Characteristics	Reaction	Characteristics	Reaction
Oxidative/fermentative	O	Acid formation from	
VogesProskauer test	−	Arabinose	+
Methyl red test	+	Saligenin	+
NO_3_^−^ reductase	+	Sorbitol	−
Growth on		Mannitol	+
At 0% of NaCl	+	Sucrose	+
At 3% of NaCl	+	Xylose	−
At 6% of NaCl	+	Raffinose	−
At 8% of NaCl	+	Glucose	+
At 10% of NaCl	+	Lactose	+
4 °C	–	Utilization of	
42 °C	+	Gluconate	−
Production of		Hydrolysis of	
Oxidase	+	Urea	+
Catalase	–	Bile esculin	+
Arginine hydrolase	+	Amylum	+

“+”, positive; “−”, negative

### Genotypic identification

**Fig. 1:**
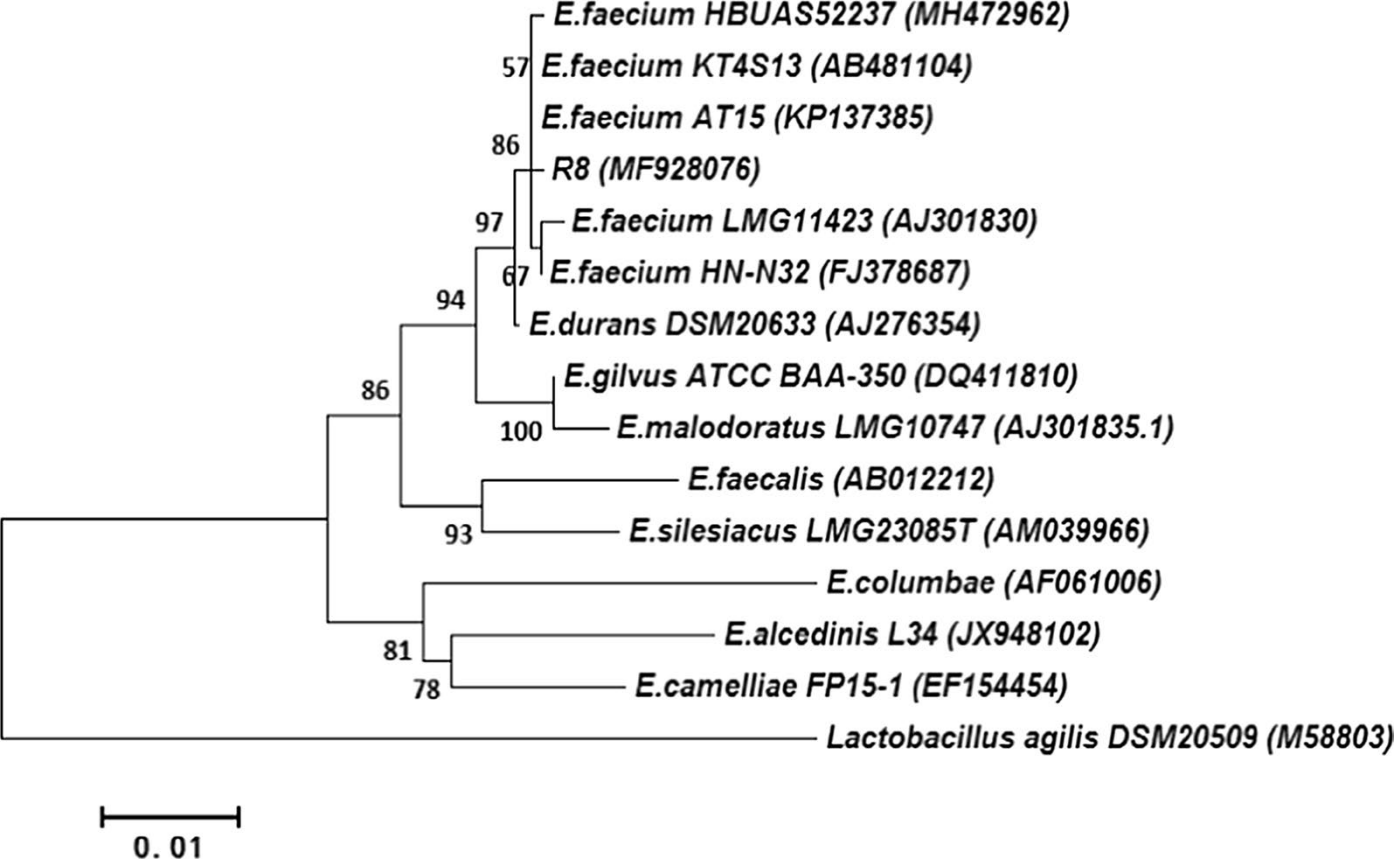
Phylogenetic tree analysis of the bacterial strain R8 based on 16S rRNA gene sequences. The R8 strain was grouped in the *Enterococcus faecium* cluster, and it was most closely related to *Enterococcus faecium* KT4S13 and AT15

The 16S rRNA gene sequence of the isolate R8 was submitted to GenBank with an accession number of MF928076. Its length was 1523 bp. The results of blast alignments indicated that R8 strain was most similar to the strain *E. faecium* KT4S13 (AB481104), *E. faecium* AT15 (KP137385), and *E. faecium* HBUAS52237 (MH472962), and their identities were 99.83%, 99.73% and 99.73%, respectively. Phylogenetic tree exhibited that the R8 strain was most closely related to the strains of *E. faecium* KT4S13 and AT15 (Fig. 1). The bacterial isolate R8 was submitted to the China General Microbiological Culture Collection Center (CGMCC) and preserved with a preservation number of CGMCC NO. 15230.

### Antagonistic activity

The isolated strain R8 inhibited all the indicator bacteria tested and the inhibition degree varied with the inhibition zones ranging from 9 to 25 mm (Table 2). It showed the most strongly inhibitory activity against *A. veronii* (25 mm), followed by *S. haemolyticus* (12 mm), *V. vulnificus* (9 mm) and *V. parahaemolyticus* (9 mm).

**Table 2:** Antagonistic activity of the R8 strain

Pathogenic bacteria	*A. veronii*	*S. haemolyticus*	*V. vulnificus*	*V. parahaemolyticus*
Bacteriostatic zone	25 mm	12 mm	9 mm	9 mm

### Haemolysis experiment and pathogenicity test

The isolate R8 was characterized as negative haemolysis. The results of challenge experiment with this isolate showed that there was no death occurring in fish from all the groups within 14 days.

### Tolerant ability against acid, bile and temperature

**Fig. 2:**
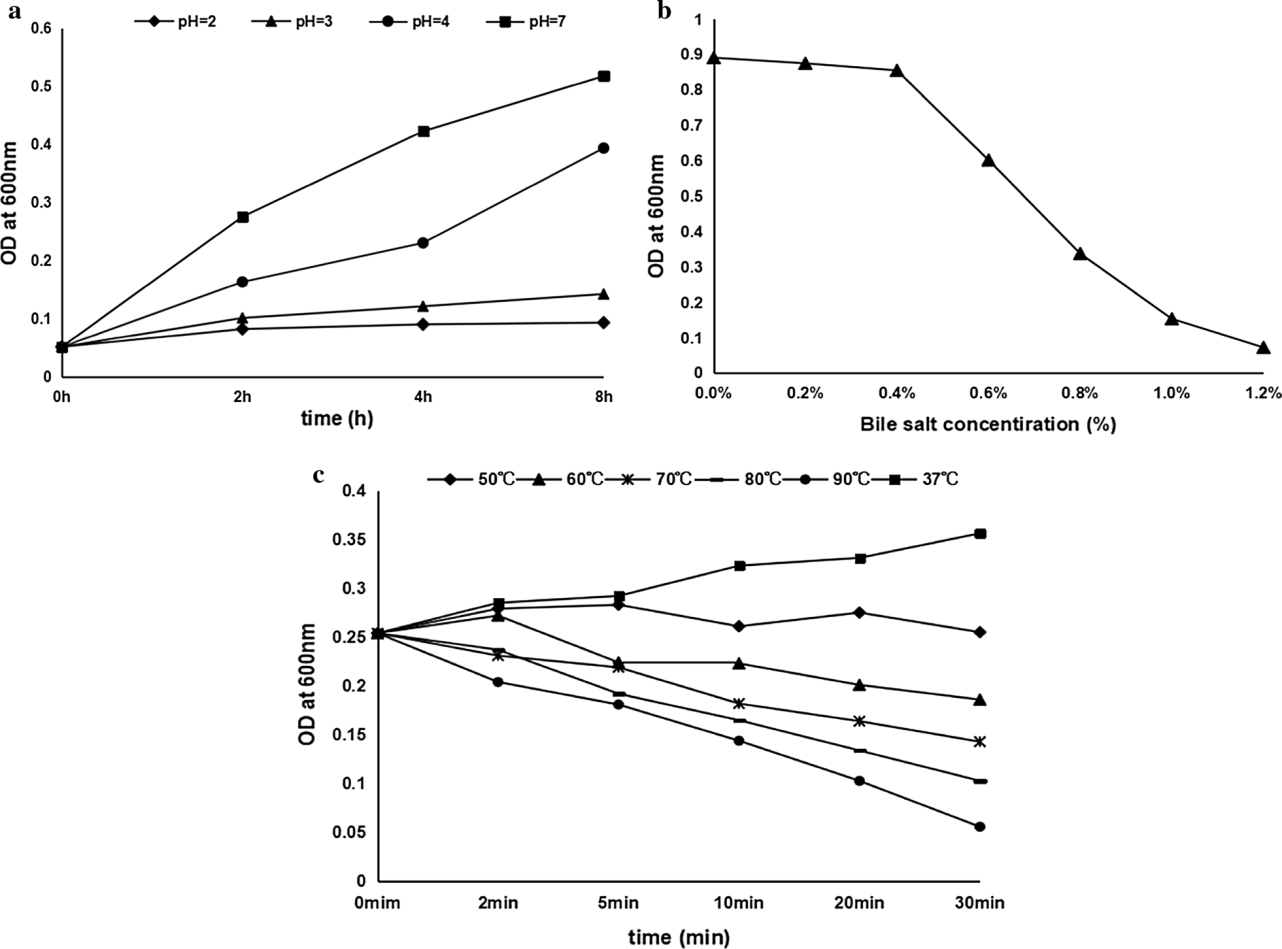
Tolerance characteristics of the bacterial strain R8. **a** Tolerance at different pH values. **b** Tolerance at different concentrations of bile salt. **c** Tolerance at different temperatures

The results of tolerant ability of the bacterial isolate R8 against acid, bile and temperature were shown in Fig. 2. This isolate could still proliferate after exposure to pH from 2.0 to 4.0 for 8 h, however the final concentrations were reduced compared to the control (pH 7.0). In addition, its proliferation rate was less affected at pH 4.0, compared with pH 2.0 and 3.0 (Fig. 2a).

Results of tolerant ability against bile salt showed that the isolate R8 could grow within the concentration range of 0.2% to 1.20% bile salts. With the increase in bile salt concentration, its growth was obviously inhibited (Fig. 2b). The results of tolerant ability of the isolate R8 against high temperature exhibited a reduction of its growth after treatment at the temperatures of 50 °C, 60 °C, 70 °C, 80 °C and 90 °C, compared to the control group (37 °C). After 30 min, the groups incubated at 90 °C had almost no growth of bacteria (Fig. 2c).

### Growth characteristics

**Fig. 3:**
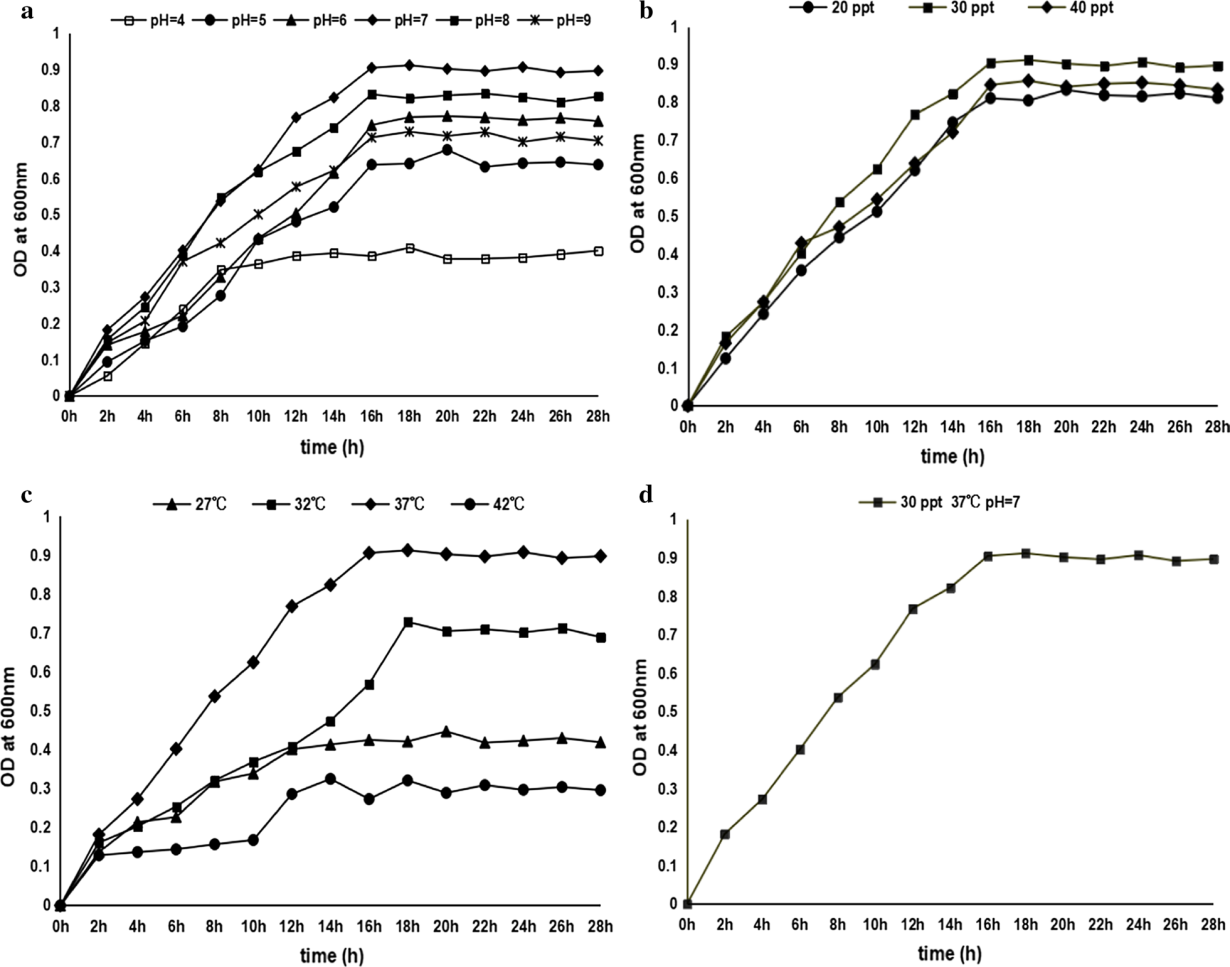
Growth characteristics of the bacterial strain R8. **a** Growth under the conditions of 20–40 ppt. **b** Growth under the conditions of pH 5.0–9.0. **c** Growth at the temperatures of 27 °C, 32 °C, 37 °C and 42 °C. **d** The growth curve of the bacterial strain R8 under the condition of pH 7.0, 37 °C, and 30 ppt showed four distinct phases of bacterial growth

The growth characteristics of the bacterial isolate R8 were shown in Fig. 3. It had the best proliferation and the highest concentration in the condition of pH value with 7.0, salinity 30 ppt and temperature 37 °C (Fig. 3a–c). Then, the growth curve was drawn under the optimal growth conditions (pH 7.0, 37 °C and 30 ppt) (Fig. 3d), exhibiting four distinct phases including latent phase (0–2 h), logarithmic phase (2–16 h), stationary phase (16–24 h) and aging phases (after 24 h).

## Discussion

The aim of this study was to search for potential probiotics in endogenous microorganisms of aquatic animals and provide a method for the screening progress. Endogenous gut microorganisms of fish antagonizing bacterial pathogens in nature are an important source for screening candidate strains of aquatic probiotics (Gómez and Balcázar 2008; Sugita and Ito 2006). To accomplish this, a bacterial strain named R8 was isolated from the intestinal tract of healthy *C. auratus* in this study. It had the key phenotypic characteristic of *Enterococcus* spp., such as growing in the presence of 6.5% NaCl and hydrolyzing bile esculin. Although *E. faecium* and *E. faecalis* are the most common species of Enterococci in animal intestines and have similar morphological and biochemical characteristics, the ability to use arabinose as carbon source of the isolate R8 could distinguish it from *E. faecalis* (Schleifer and Kilpper-Balz 1984). In order to further accurately identify this isolate, the method of 16S rDNA sequence analysis was used in this study. As expected, the isolate R8 grouped in the *E. faecium* bunch and was most closely related to *E. faecium* species.

In vitro bacteriostasis is an important indicator to evaluate whether the selected probiotics can play a role in cultured animals (Verschuere et al. 2000). Previous studies have shown that *E. faecium* exhibited the antagonism due to its adhesion to the intestinal sites, subsequently inhibiting the colonization and growth of pathogenic microorganisms. Gopalakannan and Arul (2011) found that *E. faecium* effectively resisted the infection of *A. hydrophila*, and reduced the occurrence of hemorrhagic septicemia. Swain et al. (2009) reported that *E. faecium* effectively antagonized *V. harveyi* and improved the survival rate of *Penaeus monodon*. In this study, four indicator bacteria were selected for the test of the antagonistic activity of the isolated strain. Among them, *A. veronii*, *V. vulnificus* and *V. parahaemolyticus* are the common pathogenic bacteria causing disease in aquatic animals (Uzun and Ogut 2015; Soto-Rodriguez et al. 2015). *S. haemolyticus* is mainly reported as an important nosocomial pathogen, however recently it has been frequently isolated from fish products, posing a potential health risk to consumers (Regecová et al. 2014; Sergelidis et al. 2014). The findings that the *E. faecium* R8 showed an obvious effect of inhibiting pathogenic bacteria may contribute directly or indirectly to developing the prevention treatment against infections caused by these pathogens.

Investigating the pathogenicity of a candidate probiotic strain is one of the basic criteria before its application. The hemolysis test can be used to screen the safety of strains simply, quickly and efficiently (Schulze et al. 2006), while the challenge experiment with the tested bacterial isolate will provide more accurate evaluation on its pathogenicity. In this study, the findings of the negative result in the hemolysis test and no death occurring in the challenge experiment confirmed that this *E. faecium* strain had no pathogenicity to the crucian carp.

The tolerant ability against acid and bile is important for a probiotic strain to survive and colonize in the fish gastrointestinal tract (Pérez-Sánchez et al. 2011; Sica et al. 2012). However, there is still no consensus about their precise concentrations to which the selected strains should be tolerant (Nikoskelainen et al. 2001). According to a previous report on physiological bile concentration in the fish intestine (Balcázar et al. 2008), the concentrations of bile salt from 0.2 to 1.2% were selected for investigating the bile tolerant ability of the *E. faecium* R8 in this study. When the fish stomach is filled with food, the pH values of the chyme can reach at 3.0–4.0 (Sugiura 2006; Lavelle and Harris 1997). Herein, to examine the tolerant ability against strong acid, low pH values from 2.0 to 4.0 were selected. In addition, the high temperature tolerance of a probiotic strain is an essential trait since it is often used as forage additive.

Exploring the growth characteristics and optimal growth conditions of probiotic strains is obligatory before they can be used in large-scale industrial production. Herein, the optimal growth conditions of the *E. faecium* R8 are comparable to the previously isolated *E. faecium* strains for probiotic use from Malaysian non-broiler chicken (Yusuf and Abdul-Hamid 2012) and mozzarella cheese (Nascimento et al. 2019). Although the growth of *E. faecium* R8 was more or less affected by pH value, salinity and temperature, its proliferation occurred after exposure to various growth conditions such as at pH values from 2.0 to 4.0 for 8 h, bile concentrations from 0.2 to 1.2% and high temperature of 80 °C. These findings indicate that this *E. faecium* strain has a considerable environment adaptability, being to increase the probability of growing and colonizing in the fish gastrointestinal tract when it is used as the additive probiotic. Especially, this strain having a short incubation period observed from its growth curve is beneficial to colonizing in the intestinal tract, subsequently dominating initially in the intestinal tract (Vine et al. 2004).

In summary, the *E. faecium* R8 strain from the intestinal tract of healthy *C. auratus* in the present study had probiotic properties, exhibiting inhibitory activity against bacterial pathogens, and strong tolerance to environment factors. This study provided a procedure for screening aquatic probiotics, as well enriched the species of candidate strains for aquatic probiotics.

## Data Availability

All data are fully available without restriction.

## Abbreviations

*C. auratus*: *Carassius auratus*
*E. faecium*: *Enterococcus faecium*
*A. veronii*: *Aeromonas veronii*
*S. haemolyticus*: S*taphylococcus haemolyticus*
*V. parahaemolyticus*: *Vibrio parahaemolyticus*
*V. vulnificus*: *Vibrio vulnificus*
*E. faecalis*: *Enterococcus faecalis*
FBW: Final body weight
DWG: Daily weight
MRS: de Man-Rogosa-Sharpe
LB: Luria–Bertani
DNA: Deoxyribonucleic acid
16S rDNA: 16S ribosomal DNA
16S rRNA: 16S ribosomal RNA
PCR: Polymerase chain reaction
CGMCC: China General Microbiological Culture Collection Center
CFU: Colony-forming unit
DO: Dissolved oxygen
ppt: Parts per thousand
nm: Nanometers
w/v: Weight per volume
min: Minutes
s: Seconds
g: Gravity
mL: Milliliters
μL: Microliters
L: Liter
mm: Millimeter
cm: Centimeter
g: Grams
bp: Base pairs
h: Hour
d: Day
°C: Degrees centigrade
OD: Optical density
pH: Hydrogen ion concentration
27F: 27 forward
1492R: 1492 reverse
